# Impedance Testing in Esophageal Atresia Patients

**DOI:** 10.3389/fped.2017.00085

**Published:** 2017-04-25

**Authors:** Maheen Hassan, Hayat Mousa

**Affiliations:** ^1^Department of Pediatric Gastroenterology, University of California San Diego, San Diego, CA, USA

**Keywords:** esophageal atresia, gastroesophageal reflux, esophageal pH, impedance, tracheoesophageal fistula, multichannel intraluminal impedance, pH impedance

## Abstract

Esophageal atresia patients are predisposed to gastroesophageal reflux as a result of the altered esophageal anatomy and motility. These patients experience significant morbidity from gastroesophageal reflux. As a result, an effective way to diagnose and monitor for reflux is crucial. pH-metry is able to quantify acid burden, ensure that acid suppression is adequate during long-term follow-up, and correlate acid reflux to symptoms. pH with impedance is additionally able to detect non-acid reflux as well as volume clearance, both of which also correlate with patient symptoms. It is also able to correlate extra-gastrointestinal symptoms to reflux, which may help guide treatment. If complications associated with uncontrolled reflux are identified, aggressive reflux management is necessary, oftentimes requiring surgical intervention.

Gastroesophageal reflux disease (GERD) is the most common long-term complication of esophageal atresia (EA)–tracheoesophageal fistula (TEF), affecting between 22 and 75% of pediatric patients (1–3). The increased risk of GERD in this group is due to both intrinsic dysmotility and structural factors. Primary dysmotility is a result of abnormal development of esophageal smooth muscle, with histopathologic features including distortion of smooth muscle, fibrous tissue in between smooth muscle layers (4), and tracheobronchial remnants present in the esophagus (5). In addition, there is abnormal congenital neural innervation of the esophagus, with a hypoplastic Auerbach plexus (6) and decreased interstitial cells of Cajal (7). Structurally, after surgical repair, most EA patients lose some function of the anti-reflux barrier. While anatomic changes vary from patient to patient, those most affected are patients with long gap EA. With gastric pull-up surgery, the lower esophageal sphincter (LES) is displaced proximal to the hiatus formed by the crural diaphragm, and without the overlap between the two, the anti-reflux barrier becomes incompetent (8). Gastric content can get trapped in the sac created between the LES proximally and the crural diaphragm distally, and can reflux up during swallow-induced LES relaxation. Surgical mobilization of the lower esophagus also weakens the phrenoesophageal ligament, and decreases the angle of His, further affecting the anti-reflux barrier (9).

## Complication Risks Secondary to Gastroesophageal Reflux

Given how common reflux is in EA patients and the high-risk of complications associated with GERD, it is imperative to diagnose and manage GERD appropriately in this population. Complications include dysphagia, esophagitis, Barrett’s esophagus, stricture formation, silent aspiration, and failure to thrive (Table 1). GERD is a frequently reported symptom in children and adolescence, with symptoms of GERD being reported in 22–58% of these patients (2, 3, 10, 11). As the majority of reflux experienced in these patients is acidic in nature, chronic acid exposure leads to esophagitis, increased risk of Barrett’s esophagus, and increased risk of recurrent anastomotic strictures (12–15).

**Table 1:** **Complications of gastroesophageal reflux experienced by esophageal atresia patients (10, 11, 16–18)**.

	Percentage
**Esophageal**	
Dysphagia	40–72
Esophagitis	25–53
Barrett’s esophagus	1–11
Esophageal stricture	18–50
Feeding difficulty	6–52
**Extraesophageal**	
Cough	39–80
Chronic lung disease	11
Worsening airway reactivity	13–35
Recurrent lower respiratory tract infection	13–60
Brief responsive unexplained events	Up to 53

While strictures can contribute to symptoms of dysphagia and feeding difficulties, they can also atypically present with pulmonary symptoms such as cough, chest pain, and hoarseness (16). Brief resolved unexplained events (BRUE), formerly known as apparent life-threatening events, are thought to result from either aspiration, with reflux contents from the proximal esophagus entering the larynx, or from GER in the lower esophagus stimulating respiratory symptoms (17). Further aerodigestive complications resulting from GER include chronic aspiration pneumonia, chronic lung disease with bronchiectasis and increased oxygen requirement, worsening of tracheomalacia and airway reactivity, and persistent atelectasis (13). Supporting the association between GERD and pulmonary complications is a study that demonstrated increased risk of chest infections in EA patients with early strictures compared to those without (19). Aspiration and respiratory problems can contribute to feeding difficulties in EA.

## Diagnostic Methods

### Diagnosing Gastroesophageal Reflux by Quantifying Acid Exposure

The utility of diagnosing GERD accurately and tailoring treatment accordingly is necessary to prevent the complications mentioned above. These patients are in a high-risk category given they have increased GER and almost universally have esophageal dysmotility (20, 21), which can impair reflux clearance. pH probe testing, pH-impedance testing, and wireless pH testing are currently the best objective measures for quantifying esophageal reflux (12), with each modality having its own benefits and limitations (Table 2). Twenty-four hours esophageal pH monitoring measures the frequency and duration of esophageal acid reflux, thereby quantifying esophageal acid burden. A drop in intraesophageal pH <4 for more than 5 s is considered acidic exposure (22). The reflux index (RI) is the percentage of time during the entire recording time with pH <4, with RI >7% considered abnormal, an RI <3% considered normal, and an RI between 3 and 7% indeterminate (22). While the sensitivity of abnormal esophageal pH in predicting erosive esophagitis in adults and children is high, ranging from 83 to 100% (23, 24), there are limitations to standard pH monitoring. It is a poor detector of weakly acidic (pH 4–7) reflux (25) and can also overestimate acid exposure by picking up “pH-only” episodes, in which there is no detected retrograde liquid refluxate (26). In infants and children, weakly acidic GER is more prevalent than in adults (26, 27), which can explain why symptoms are not always detected by esophageal pH monitoring (23). This elucidates the limitation in depending on only pH monitoring to diagnose reflux.

**Table 2:** **Benefits and limitations of pH-only versus pH-impedance testing (23, 25, 28, 29)**.

	Benefits	Limitations
pH-only	Quantifies frequency and duration of acid exposure Measures chemical clearance Able to correlate acid reflux to symptoms Readily available Easier to interpret than pH-impedance	Unable to detect non-acid and weakly acid reflux Can overestimate acid exposure by picking up “pH-only” episodes Limited utility in patients on acid suppression, continuous feeds, or frequent feeding schedule
pH-impedance	Quantifies acid and non-acid reflux Detects liquid, gas and mixed refluxate Measures volume and chemical clearance Quantifies the height of refluxate	Analysis is time consuming Low baseline impedance in esophageal atresia patients makes it difficult for automated analysis to detect reflux events, and must be manually reviewed Limited availability in certain medical centers and practices

### Utilizing Multichannel Intraluminal Impedance (MII) in the Diagnosis of Gastroesophageal Reflux

Multichannel Intraluminal Impedance is an alternative diagnostic tool that utilizes change in impedance to measure the anterograde and retrograde movement of fluid, solids, and air in the esophagus. Dual pH-multichannel intraluminal impedance (pH-MII) is able to detect both acid and non-acid GER, detect anterograde versus retrograde flow thereby distinguishing between swallows and GER, determine the height of refluxate, and differentiate between liquid, gas, or mixed refluxate (28, 30). MII also provides information about bolus transit, duration of bolus presence, time of bolus clearance, and time of acid clearance.

Though initially used as a research tool, pH-MII has been shown to be very useful in assessing reflux and clearance. The definitions listed below have been established based on several studies (26, 31):

- Liquid reflux: drop in impedance to ≤50% of the baseline value, with subsequent recovery, in two or more of the distal-most channels.
- Acid reflux: liquid reflux (using the aforementioned definition) in which the pH decreases and remains <4 for ≥5 s.
- Non-acid reflux: liquid reflux (using the aforementioned definition) in which the pH increases, is unchanged, or decreases by at least 1 pH unit while maintaining pH ≥4.
- Gas reflux: simultaneous and rapid increases in impedance (>3,000 Ω) in two or more of the distal-most channels.
- Proximal reflux/“high reflux”: the refluxate reaches either/or both of the most proximal channels.
- Distal reflux: the refluxate is confined to the two most distal impedance channels.
- Bolus clearance time (BCT): time for bolus clearance from the esophagus.
- Acid clearance time (ACT): time for acid clearance from the esophagus.

Reference values for reflux parameters in infants and children on pH-MII over a 24 h period were previously published by a multicenter study (Table 3) (32). Patients were selected based on having no evidence of acid reflux or symptoms associated with regurgitation, off anti-reflux medications at the time of the procedure, and no fundoplication. Based on the study findings, the following would be considered abnormal over a 24 h period, as it is above the 95th percentile in this selected group of infants and children: >48 acid reflux episodes or more than 67 non-acid reflux episodes in an infant; >55 acid reflux episodes or >34 non-acid reflux episode in a child; >93 total GER events in an infant and >71 total GER events in a child. A limitation in this study, as well as all studies done in children, is in the ethics of performing pH-MII in asymptomatic children. As all patients were symptomatic, true normal values cannot be established. However, by setting strict selection criteria, it is likely that the patients selected have physiologic GER and can be used as a reference. Reflux parameters have also been described by a prior large-scale study of 700 children (33). Patients with normal RI had a mean of 39 ± 31 reflux episodes, compared to patients with pathological RI that had a mean of 58 ± 43 reflux episodes. The children selected were all symptomatic, with 21% having documented acid reflux. In this study, reflux was not differentiated into non-acid and acid reflux. A study to establish normal reflux parameters was additionally done in preterm infants, who were otherwise healthy (34). This study was limited due to all patients being on tube feedings, which can affect the number of reflux episodes, as the tube stents open the LES.

**Table 3:** **Normal values for reflux on pH-multichannel intraluminal impedance per 24 h in infants and children**.

	Infants		Children	
	Median (IQR)	95th %	Median (IQR)	95th %
Index of acid regurgitation (%)	0.6 (0.3–0.9)	1.4	0.4 (0.2–0.8)	1.3
Number of acid regurgitation episodes in 24 h	20 (11–26)	48	14 (11–15)	55
Index of non-acid regurgitation (%)	0.7 (0.5–1.2)	2.5	0.1 (0–0.3)	1
Number of non-acid regurgitation episodes in 24 h	32 (16–45)	67	6 (3–11)	34
Index of GER episodes (%)	1.4 (0.9–1.2)	2.9	0.6 (0.3–1.2)	2.4
Number of GER episodes in 24 h	54 (33–69)	93	21 (11–41)	71
Mean GER bolus clearance time (s)	13 (11–16)	20	15 (12–19)	32

*Adapted from Mousa et al. (32)*.

### pH-MII in Determining Esophageal Clearance

pH-multichannel intraluminal impedance is useful in calculating esophageal clearance. Volume clearance involves primary and secondary peristalsis, and is followed by chemical clearance that neutralizes acid. The efficiency of volume clearance of a reflux episode is generally assessed using the most distal impedance channel. The presence of reflux is identified by the impedance waveform dropping to 50% of the baseline. The bolus is cleared from the distal esophagus when the impedance waveform again reaches 50% of impedance baseline (35). Reference values have been published, with the upper 95th percentile of bolus clearance being 20 s in infants and 32 s in children (Table 3).

While volume clearance is known to be accomplished by esophageal peristalsis, chemical clearance is known to be accomplished primarily by bicarbonate-rich saliva that neutralizes acid and washes the esophageal walls of gastric and duodenal debris (36). Chemical clearance is defined as the duration of esophageal acidification immediately followed the end of volume clearance (37). It begins the moment the impedance waveform in the distal-most channel returns to 50% of baseline and ends when the pH waveform reaches pH 4. Physiologic norms were determined for infants up to 1 year of age, and children between ages 1 and 18 years (38): the upper 95th percentile of physiologic chemical clearance duration was 148.5 s for infants and 114.4 s for children. These children had no fundoplication, no positive reflux-symptom associations, were not taking anti-reflux medications at the time of the study, and had acid gastroesophageal reflux indices ≤3% for the children and ≤6% for the infants.

Clearance, particularly of non-acid reflux, cannot be picked up by pH testing alone. Comparing infants with EA and controls with GER, the mean ACT and mean BCT are significantly longer in the EA group (39). Correlation between symptoms and clearance time in the EA group showed that the median ACT and BCT were significantly shorter in patients without symptoms than in those with symptoms. This suggests that it is not the acidity of the reflux is that influences symptoms, but the clearance. Findings of prolonged bolus and ACT were similarly found in older children (40). Studies have shown that esophageal clearance can indicate the severity of esophageal dysmotility. In one study, while 79% of swallows were accompanied by abnormal motility patterns, approximately 60% of swallows showed abnormal bolus transit, and 66% of all GER episodes initiated no clearing mechanism (41). Furthermore, EA patients have a significantly lower percentage of complete bolus transit for liquid and viscous swallows, and their higher bolus index and reflux indices are significantly related to increased symptom scores (29).

### Correlating Symptoms with Reflux

Three indices are used to quantify the temporal relationship between GER and symptoms. Though they have been validated in adults, currently there are few studies validating the use in children (33, 42, 43).

- Symptom index (SI): the number of symptoms associated with reflux divided by the total number of symptoms in 24 h. SI >50% is considered abnormal.
- Symptom sensitivity index (SII): the number of symptoms associated with reflux divided by the total number of reflux events in 24 h. SII >10% is considered abnormal.
- Symptom associated probability (SAP): calculation of statistical relation between reflux and symptoms using Fisher’s exact test. SAP >95% is interpreted as good temporal association between GER and the recorded symptom.

The SI and SSI are simple to calculate, with the former being used to determine the percentage of symptoms that are associated with reflux events and the latter used to determine the percent of reflux events associated with symptoms (30). The SI does not take into account all reflux episodes and can provide false-positive results when the number of reflux episodes is large or the number of symptoms is small. The SSI does not take into account the total number of symptoms and can result in false positives when the number of reflux episodes is small or the number of symptoms is small. The SAP takes all parameters into account and is the strongest statistical parameter for symptom association analysis. The minimal number of symptoms to obtain an accurate and reliable SAP is uncertain (27, 44). Positive symptom association, which suggests causality between reflux and symptoms, is defined when both SI and SSI are positive or when SAP is positive. Further limitations to these symptom indices include (44): (1) registration of symptoms in a timely fashion is dependent on the child and/or parent. (2) The time interval of 2 min between a reflux event and symptom is the accepted interval, based on consensus, to demonstrate a time association. However, this time interval is not evidence based and may differ based on symptoms. For some symptoms that have a chronic GER relation such as wheezing, laryngitis, or bronchial hyperreactivity, temporal symptoms association may not be achieved. Symptoms such as cough, apnea, and chest pain likely have a shorter time frame.

pH-multichannel intraluminal impedance monitoring is useful in evaluating and correlating non-acid reflux with symptoms in the following patient groups: symptomatic patients on proton pump inhibitor (PPI), patients on continuous feeds, patients with extraintestinal symptoms, patients with BRUE, and GER symptoms with normal pH probe and endoscopy results (22). Since many EA patients are already on anti-reflux therapy, may have a frequent feeding schedule, or may be on tube feeds, the majority of their refluxate is non-acidic and would otherwise be missed by conventional pH testing (28). In a study comparing infants with EA and controls with GER, reflux events in both groups were mainly non-acid (39). Weakly acid reflux has also been shown to be responsible for a significant percentage of symptoms in EA patients (29). In a separate study comparing EA patients and controls with GER, there was no difference in the total retrograde bolus movements between the two groups, though the EA group had significantly higher non-acid RI (21, 29). In the EA patients, 28–42% of symptom occurrences were associated with retrograde bolus movements. The utility of pH-impedance compared to pH-metry alone is further elucidated when comparing the SI between the two modes. Significantly more EA patients had a positive SI when using pH-MII than pH probe alone (42).

pH-impedance is useful in quantifying the proportion of reflux reaching up to the proximal esophagus, referred to as “high reflux.” EA patients frequently experience extraesophageal symptoms, and pH-MII has the unique ability to determine if these symptoms correlate with reflux episodes, whether they are acid or non-acid. In one study, 39% of the coughs recorded were associated with reflux (29). In that study, of the four patients who showed more than 50% high-reflux episodes, three had chronic pulmonary problems with frequent postprandial coughing. Of these high refluxes, 47% were weakly acidic and 53% were acidic. In another study, 62% of coughs were associated with reflux in children ≤1 year old, and 58% of coughs were associated with reflux in children >1 year old (39). Cough episodes were more commonly seen with acid reflux, though compared to children >1 year old, younger children had more frequent cough episodes related to non-acid reflux. In a study correlating symptoms in EA to the presence of GERD, the most frequent symptoms in children with GERD included cough, recurrent bronchitis, and heartburn, though this did not reach clinical significance (45).

When patients with EA have non-acid reflux associated with complications, particularly pulmonary or stricture related, medical management with prokinetics is recommended. If this fails, fundoplication or transpyloric feeds should be considered. While fundoplication can have higher complications in EA patients, it is indicated in the following cases: patients with significant extraesophageal symptoms related to GERD including cyanotic spells, patients with recurrent anastomotic strictures, and esophagitis despite maximal PPI therapy (12).

## Benefits of pH-MII

As discussed above, pH-MII provides multiple benefits over pH probe alone, and should be considered, when available, for diagnosing and monitoring for GER in EA. It not only quantifies acid and non-acid exposure, but also is more effective in correlating symptoms to reflux, and can measure both volume and chemical clearance. In a pH-MII study of 700 children with GERD symptoms, 45% of the patients with abnormal GER would not have been recognized by 24-h pH measurement alone (33). In addition, extraintestinal symptoms of GER, which were more common in younger children, were more often correlated with pH-MII as compared to pH alone.

It should be noted that GERD is often more severe than predicted based on the clinically reported symptoms (21, 29). Reliance on symptoms is insufficient in determining whether acid suppression is needed. Given the high-risk of complications from GERD, particularly the high-risk of anastomotic stricture in the first year of life, it is recommended that patients stay on empiric therapy with acid suppression for the first year of life. They should also undergo monitoring of GER with impedance/pH and/or endoscopy at the time of antacid discontinuation and during long-term follow-up (12).

## Limitations of pH-MII

One of the limitations of pH-MII in patients with EA is that the baseline impedances are 75% lower than control patients (29). Because of this, software analysis often does not detect reflux events. As a result, manual analysis must be done in addition to automated, to prevent underreporting of reflux (12). While there is a single large study that has reported age-related normal data for reflux indices (32), symptom association statistics are largely based on adult data. The time interval between symptoms and reflux events is based on consensus, with little evidence on the ideal time frame between different types of symptoms. As there are no large outcomes, studies on treating weakly acid and non-acid reflux with anti-reflux surgery, medications that decrease transient LES relaxation, or promotility agents, the clinical relevance of measuring this type of reflux remains debatable. In addition, the analysis of pH-MII requires special training and it is time consuming. These tests are also not available to every medical practice. pH probe testing is easier to interpret and more accessible to providers.

## Conclusion

Because of the increased prevalence and significant morbidity associated with GER in EA/TEF patients, diagnosing and monitoring for GER is essential. The recommendations are to treat all EA patients in the first year of life with PPIs, and to monitor for GER thereafter. pH monitoring is recommended to evaluate the severity of acid reflux and the symptoms associated with it. pH-impedance monitoring provides additional benefits of correlating non-acid reflux and esophageal clearance with symptoms. These tools help guide the duration of antacid therapy and the need for surgical intervention.

## Author Contributions

MH is the primary author, and she drafted the paper. HM is the supervisor and corresponding author, and she reviewed and edited the paper.

## Conflict of Interest Statement

The authors disclose no conflict of interest. This review was conducted in the absence of any commercial or financial relationships that could be construed as a potential conflict of interest.
